# CTLA-4 +49 G/A, a functional T1D risk SNP, affects CTLA-4 level in Treg subsets and IA-2A positivity, but not beta-cell function

**DOI:** 10.1038/s41598-018-28423-9

**Published:** 2018-07-04

**Authors:** Yang Chen, Shu Chen, Yong Gu, Yingjie Feng, Yun Shi, Qi Fu, Zhixiao Wang, Yun Cai, Hao Dai, Shuai Zheng, Min Sun, Mei Zhang, Xinyu Xu, Heng Chen, Kuanfeng Xu, Tao Yang

**Affiliations:** Department of Endocrinology, The First Affiliated Hospital of Nanjing Medical University, Nanjing, 210029 China

## Abstract

To investigate whether CTLA-4 +49 G/A (rs231775), a tagSNP in Asian, is a functional T1D SNP, we genotyped this SNP with 1035 T1D patients and 2575 controls in Chinese Han population. And 1280 controls measured insulin release and sensitivity based on an oral glucose tolerance test; 283 newly diagnosed T1D patients assayed C-peptide level based on a mixed-meal tolerance test. 31 controls were analyzed for different T cell subsets by multi-color flow cytometry. Under additive model, we found that CTLA-4 +49 G/A was significantly associated with T1D (P = 2.82E-04, OR = 1.25, 95% CI: 1.12–1.41), which was further confirmed by meta-analysis (P = 1.19E-08, OR = 1.65, 95% CI: 1.38–1.96) in Chinese Han population. Although we did not find any association between this SNP and beta-cell function in either healthy individuals or newly diagnosed T1D patients, healthy individuals carrying GG/GA genotypes had lower CTLA-4 expression in naïve or activated CD4 Treg subsets (P = 0.0046 and 0.0317 respectively). A higher positive rate of IA-2A was observed among T1D patients with GG genotype compared with AA (OR = 0.51, 95% CI: 0.30–0.84, p = 0.008). Collectively, CTLA-4 +49 G/A reached a GWAS significant association with T1D risk in Chinese Han population, affects CTLA-4 expression in Treg subsets and subsequently humoral immunity in T1D patients.

## Introduction

Cytotoxic T lymphocyte-associated antigen-4 (CTLA-4, also named CD152), is homologous to CD28 and belongs to the immunoglobulin superfamily. As a negative regulator of T cell responses, CTLA-4 constitutively expressed on Treg cells and upregulated on activated conventional T cells (Tconvs)^1,2^. Most of the CTLA-4 protein resides intracellularly in T cells, only a small fraction is expressed on T cell surface and engaged to its ligands B7–1 or B7–2. Studies have indicated that CTLA-4 exerts its function through both cell-intrinsic and cell-extrinsic mechanisms. Recently, CTLA-4 has been considered as a clinically exploited immune biomarker^3,4^. For the stability of Tregs, expression of CTLA-4 keeps hyporesponsive and suppressive function *in vivo*^5–9^. Dysregulated expression of CTLA-4 leads to immune homoeostasis imbalance and autoimmunity both in mouse and human^10,11^.

Common single nucleotide polymorphisms (SNPs) in CTLA-4 have been found to have a role in susceptibility to autoimmune diseases in different populations, such as type 1 diabetes, celiac disease and rheumatoid arthritis^12–15^. Previous study reported that CT60 (rs3087243) in the 3′-UTR of CTLA-4 gene was identified as the principal marker for genetic risk^14^, and CTLA-4 +49 G/A (rs231775), another most studied tagSNP in Asians with moderate LD (r^2^ = 0.55) to CT60, causes an amino acid exchange (threonine to alanine) and has been shown to be associated with several autoimmune diseases, including type 1 diabetes^12,16^. However, the significance of CTLA-4 +49 G/A and CT60 SNPs with T1D risk in Chinese Han population remains elusive, as previous studies were mostly conducted on smaller size, and hence were less powerful with conflicting findings. Furthermore, the exact functional relevance of these two SNPs remains to be characterized.

Here we investigated the possible association between CTLA-4 +49 G/A, CT60 and T1D risk in a Chinese Han population. Subsequently we assessed their impacts on glycemic traits based on an oral glucose tolerance test (OGTT) in healthy individuals and residual C-peptide levels in newly diagnosed T1D patients, and their influences on quantitative changes of CTLA-4 expression in different T cell subsets and autoantibody positivity during T1D development.

## Materials and Methods

### Subjects

The study consisted of 1035 unrelated T1D patients and 2575 healthy controls recruited from the First Affiliated Hospital of Nanjing Medical University between January 2008 and December 2016. Except for the World Health Organization (WHO) criteria, only diabetic patients with at least one autoantibody positive (ZnT8A, GADA, IA-2A or IAA) were included as T1D cases, and cases that had clinical features of latent autoimmune diabetes in adults (LADA) were also excluded. Clinical characteristics of the study population are shown in Table S1. The average age at diagnosis for T1D cases was 19.9 ± 13.3 years old and median duration of diabetes was 5.9 ± 4.6 years. And healthy controls were enrolled from the same geographical region with a mean age of 48.9 ± 12.7 years old, who did not have diabetes mellitus or overt autoimmune diseases or any chronic diseases, such as such as autoimmune thyroid diseases, alopecia areata or vitiligo, *etc*. The inclusion criteria for the non-diabetic healthy controls were as follows: (1) HbA1c ≤ 6.0%, 2) fasting plasma glucose (FPG) < 5.6 mmol/l and 2 h plasma glucose (PPG) < 7.8 mmol/l. All samples were collected with appropriate informed consent from all participants and/or their guardians in a written way, and study population was determined as Chinese Han by questionnaire. The study was approved by the Ethics Committee from the First Affiliated Hospital of Nanjing Medical University and conducted according to the principles of the Declaration of Helsinki as revised in 2000.

### Genotyping

Genomic DNA was extracted from peripheral blood lymphocytes using the QIAamp DNA extraction kit (QIAGEN, Germany). CTLA-4 +49 G/A and CT60 SNPs were genotyping by single-nucleotide extension of SNP-specific probes using a SNaPshot ddNTP primer extension kit. The extended probes were electrophoresed on an ABI Prism 3100 genetic analyzer (Applied Biosystems, Foster City, CA). The genotyping success call rate was above 97%, and 180 samples measured in duplicate (≈5%) were in complete concordance. The genotyping primers are as follows: for +49 G/A in CTLA-4, forward, 5-TCCTTGATTCTGTGTGGGTTC-3; reverse, 5-TGCTCCAAAAGTCTCACTCAC-3; Extended, 5-TTTTTTGGCTCAGCTGAACCTGGCT-3; and for CT60 SNP, forward, 5-CTTCATGAGTCAGCTTTGCAC-3; reverse, 5-CCATGACAACTGTAATGCCTG-3; Extended, 5- TTTTTTTTTCTATGTCTGTGTTAACCCA-3.

### Meta-analysis

(1) ***Search strategy*** Electronic databases (PubMed and EMBASE and CNKI) were searched up to May 2018 for all genetic association studies evaluating the CTLA4 +49 G/A or CT60 in T1D risk. Search strategies were investigated using combinations of the following search terms: (CTLA-4 or CD152) and (type 1 diabetes or T1D) and (variant or polymorphism). No restriction was set on the source of control participants (general population, clinic or hospital). To identify additional relevant studies, we also searched the reference lists and the Medline option ‘Related Articles’ of the selected articles. (2) ***Inclusion criteria**** and data extraction* Any human genetic association study, regardless of sample size, was included in the meta-analysis if it met the following criteria: a, study evaluated the association of investigated CTLA-4 SNPs with T1D risk; b, study had sufficient published data to estimate an odds ratio (OR) with 95% confidence interval (CI) or provided raw data that allowed us to calculate them; c, if the data were duplicated or had been published more than once, the most recent and complete study was chosen; d, studies were excluded if the genotype distribution of the controls deviated from Hardy-Weinberg equilibrium (HWE); e, review articles, abstracts, editorials, reports with incomplete data, and studies based on pedigree data were also excluded. All the identified studies were carefully reviewed by two investigators independently, and any discrepancies were resolved by discussion, when necessary, adjudicated by a third reviewer.

### In silico annonation for +49 G/A or CT60 in CTLA-4

We searched the GTEx v7 database (https://gtexportal.org/home/) to identify the association between +49 G/A or CT60 and changes in expression (eQTLs) with particular attention to pancreases and whole blood (containing monocyte and lymphocyte). Then we analyzed regulatory chromatin states from DNase and histone ChIP-Seq (Roadmap Epigenomics Consortium, 2015), Proteins bound in ChIP-Seq experiments (ENCODE Project Consortium, 2011) to further identify functional evidence associated with these two SNPs.

### Baseline Clinical Characteristics and OGTT in healthy individuals

Among healthy individuals, 1280 participants underwent a 75-g OGTT after a 12-h fast. Blood samples were collected to assay plasma glucose and serum insulin at fasting, 30 and 120 min based on an OGTT. Plasma glucose level was analyzed by the hexokinase method with an autoanalyzer (Modular E170, Roche); serum insulin level was measured using insulin radioimmunoassay kit (BNIBT, China). Measures of insulin sensitivity and beta cell function derived from the OGTT were calculated according to the formulas in Table S2.

### Mixed-meal tolerance test (MMTT) in new onset T1D patients

283 eligible newly diagnosed T1D patients underwent a mixed-meal tolerance test (MMTT) using standard procedures. Serum samples were assayed for C-peptide level at the time points of fasting, 60 min and 120 min. The C-peptide area under curve (AUC) was calculated using the trapezoidal method and then divided by the time period of the test. Samples with C-peptide > 0.2 nmol/L were considered to have residual beta cell function. Serum C-peptide concentrations were analyzed by Automatic electrochemical luminous instrument (Roche, Switzerland).

### Laboratory measurements for autoantibodies in T1D patients

T1D Patients were regarded as autoantibody positive if they had one or more antibodies, including ZnT8A, GADA, IA-2A, and IAA. ZnT8A, GADA, and IA-2A were measured separately by protein A radio-binding assays using ^[35S]^methionine-labelled *in vitro*-transcribed/translated recombinant human GAD65, IA-2A, and the carboxy-terminal portion of ZnT8 for the two major variants at amino acid 325, respectively. IAA was measured by ELISA (Biomerica) and only tested ≤2 weeks after diagnosis to avoid detection of antibodies to injected insulin.

### Flow Cytometry

The following reagents were purchased from BioLegend or BD Biosciences: anti-CD3 (SK7) FITC, anti-CD4 (SK3) BV786, anti-CD8 (SK1) APC-H7, anti-FoxP3 (259D/C7) PE-CF594, anti-CD25 (M-A251) PE-Cy7, anti-CD45RA (HI100) Alexa700, CCR7 (GO43H7) BV605 and anti-CTLA-4 (BNI3) BV421. Peripheral mononuclear blood cells (PBMCs) were isolated from whole blood at study entry and frozen at a core facility. After Aqua staining for cell viability, thawed PBMCs were stained with surface antigens (CD3, CD4, CD8, CD25, CD45RA and CCR7), then these cells were fixed and permeabilized according to manufacturer’s instructions (eBioscience) and further stained for intracellular FOXP3 and CTLA-4, and analyzed by flow cytometry with the BD Aria II (San Jose, CA) and Flow Jo v10 software (TreeStar, Ashland, OR).

### Definition of activated Treg, resting Treg and secreting T cells

Activated Treg (aTreg) as CD4^+^CD45RA^−^FoxP3^hi^ or CD4^+^CD45RA^−^CD25^hi^ T cells, naive Treg (nTreg) as CD4^+^CD45RA^+^FoxP3^+^CD25^+^ T cells and secreting Treg (sTreg) as CD4^+^CD45RA^−^FoxP3^+^CD25^+^ T cells.

### Statistical Analysis

The frequencies of allele and genotype of CTLA-4 +49 G/A and CT60 SNPs were assessed by direct counting. Associations between these two SNPs and T1D risk in case-control study and departures from the Hardy-Weinberg equilibrium (HWE) were analyzed by the χ^2^ test. Combined meta-analysis was performed using the Mantel-Haenszel (fixed-effects) and DerSimonian and Laird (random-effects) tests after testing for heterogeneity. The magnitude of the association of different genotypes with T1D risk was estimated using odds ratio (OR) tests with 95% CI. The false-positive report probability (FPRP) test of Wacholder *et al*.^17^ was applied to address the issue of false-positive SNP associations. Associations for glycemic quantitative traits were examined by using logistic regression analysis under the additive model. Values of serum insulin, IGI, BIGTT-AIR, CIR, HOMA-B, HOMA-IR, and ISIMatsuda were logarithmically transformed. For pairwise comparisons either student’s unpaired two-tailed t test or Mann-Whitney test were used depending on whether the data followed a Gaussian distribution or not. For more than 2 groups one-way ANOVA or Kruskal-Wallis test were used. Error bars represent mean ± SEM. Corrections for multiple comparisons were performed by the Bonferroni test. All P values were two-tailed and differences were considered statistically significant with a P value of <0.05 (*), <0.01 (**) or <0.001 (***). These statistical analyses were done using SPSS 18.0, RevMan 5.2 and GraphPad Prism 7.0 software.

## Results

### Both +49 A/G and CT60 in CTLA-4 are associated with T1D risk in a Chinese Han population

The distributions of genotypes of CTLA-4 +49 G/A and CT60 were in agreement with HWE (P > 0.05 in both T1D patients and healthy controls). As shown in Table 1, CTLA-4 +49 G/A was significantly associated with T1D risk under additive, dominant and recessive models (P = 2.82E-04, 1.58E-03 and 8.6E-04 respectively). For CT60, the results were similar to +49 G/A except for dominant model (P = 0.114). LD plots demonstrated that these two SNPs are in moderate LD (r^2^ = 0.52 in healthy individuals), which is similar to the results from 1000 Genomes Project database (r^2^ = 0.55). We further performed a combined meta-analysis, and the trial flow and genotype distributions of the identified studies are shown in Figure S1 and Table S3. As studies were not enough to perform a meta-analysis for CT60, we only assessed the association for CTLA-4 +49 G/A. Our results indicated that +49 G/A was associated with T1D risk at a GWAS significance (P = 1.19E-08, OR = 1.65, 95% CI: 1.38–1.96) (Figure S2). The P-values of Egger’s test was 0.132, and the FPRP value remained below 0.2 even for a prior probability of 0.0001. These confirmed that our results were quite robust.

**Table 1:** Genotype distributions and associations of CTLA-4 +49 G/A and CT60 SNPs with T1D risk in Chinese Han population.

SNP	pos (hg38)	Ref/Alt	Genotype distribution		MAF		Additive		Dominant		Recessive	
			T1D	Controls	T1D	Controls	OR (95%CI)	P value	OR (95%CI)	P value	OR (95%CI)	P value
+49 G/A	203867991	A^**§**^/G	73/403/529	268/1086/1178	0.273	0.320	1.25 (1.11–1.41)	2.82E-04***	0.78 (0.68–0.91)	1.58E-03**	1.28 (1.10–1.48)	8.60E-04***
CT60	203874196	G/A^**§**^	729/248/28	1725/749/100	0.151	0.184	0.79 (0.68–0.91)	1.09 E-03**	1.41 (0.92–2.16)	0.114	0.77 (0.65–0.90)	1.02 E-03**

Note: ^**§**^MAF, minor allele frequency. **P < 0.01 or ***P < 0.001.

### Both +49 A/G and CT60 in CTLA-4 are putatively functional

To evaluate how +49 G/A and CT60 may confer disease susceptibility, we explored the genetic and regulatory architecture of thses two SNPs. Base on GTEx database, the only eQTL for +49 G/A reaching genome-wide significance is in testis (p = 1.0E-7). Although many other tissues have less significant p values, the eQTL for this SNP is in the same direction as for testis, and the overall meta analysis of all tissues is p = 6E-16. Interestingly, the most significant association in searching on eQTLs for the CTLA-4 gene is with CT60 in testis (p = 7.8E-12). Among them, both +49 G/A and CT60 did show moderate association in spleen and is in the same direction as testis (P = 0.034 and 0.0026 respectively), but they did not associate with eQTL in either the pancreas or whole blood (P > 0.05). Using ChIP-Seq experiments from the ENCODE datasets, we found that MAX and STAT3 binding to CT60, but not +49 G/A. In addition, according to Roadmap Database, both +49 G/A and CT60 acted as an enhancer for H3K4me1 and H3K27ac, and a promoter for H3K4me3 in different T cell subsets, including Treg, Th17, etc (Table S4). Taken together, these data indicate that these two SNPs may be functional with regulatory implications.

### Neither +49 A/G nor CT60 in CTLA-4 has any effect on residual C-peptide in newly diagnosed T1D patients or islet function in healthy individuals

**Figure 1:**
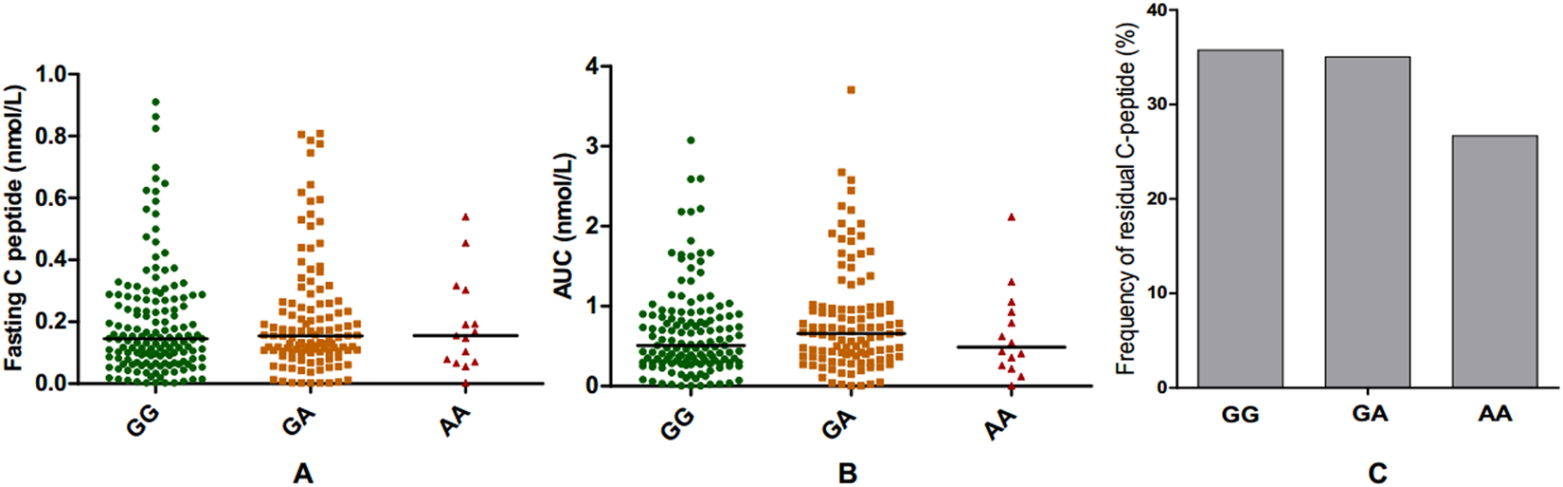
Associations of CTLA-4 +49 G/A with fasting C-peptide, C-peptide AUC and residual C-peptide in newly diagnosed T1D patients. Note: (**A**–**C**) Associations between +49 G/A and fasting C-peptide, C-peptide AUC and residual C-peptide respectively. GG = 151, GA = 117, AA = 15; fasting C-peptide and AUC were adjusted by age, gender, age at T1D onset and duration; residual C-peptide was defined as Fasting C-peptide ≥0.2 nmol/L.

To evaluate potential *in vivo* effects caused by these two SNPs, we first examined whether they affect residual β-cell function in newly diagnosed T1D patients. As shown in Fig. 1A,B, after adjustment for gender, age at diagnosis and disease duration, +49 G/A did not associate with fasting C-peptide or AUC of C-peptide in 283 newly diagnosed T1D patients (P = 0.528 and 0.439 respectively). And none of the risk GG carriers display any decline in residual β-cell function compared with 27% and 35% of the AA and GA carriers, respectively (P = 0.780), as shown in Fig. 1C. We also investigated whether +49 G/A affects β-cell function in 1280 clinically well-characterized healthy individuals based on OGTT. As shown in Table 2, after adjustment for age, sex and BMI, this SNP had no association with plasma glucose level or insulin level, except for a marginal association with plasma glucose 30 min post OGTT (P = 0.055). Furthermore, this SNP did not exhibit any convincing association with either fasting insulin derived indexes (HOMA-β, HOMA-IR) or insulin release and insulin sensitivity indexes derived from an OGTT (all P > 0.05). For CT60, the results was similar to +49 G/A, as shown in Table S5 and Figure S3. Taken together, the results suggested these two SNPs in CTLA-4 gene had no observed effect on islet function.

**Table 2:** Associations of CTLA-4 49 G/A With Quantitative Traits in 1280 Glucose-Tolerant East Chinese Individuals.

	AA	GA	GG	β	SE	P	adjusted P
n (men/women)	128(45/83)	568(166/402)	579(186/393)				
Age (years)	55.9 ± 9.4	56.0 ± 9.5	55.6 ± 9.0				
BMI (kg/m2)	23.8 ± 5.5	23.4 ± 3.0	23.3 ± 3.2	0.176	0.146	0.228	
**Plasma glucose (mmol/l)**							
Fasting	5.3 ± 0.3	5.3 ± 0.3	5.3 ± 0.4	−0	0.015	0.999	0.807
30 min post OGTT	8.6 ± 1.6	8.6 ± 1.4	8.8 ± 1.5	−0.12	0.062	0.092	**0.055**
120 min post OGTT	6.2 ± 0.9	6.1 ± 1.0	6.2 ± 1.0	−0.04	0.04	0.472	0.287
**Serum insulin (mIU/l)**							
Fasting	9.23(7.33–13.45)	10.12(7.25–14.00)	9.72(7.33–13.61)	0	0.01	0.806	0.967
30 min post OGTT	52.59(38.11–80.91)	59.70(40.59–95.21)	56.39(36.53–87.34)	0	0.013	0.807	0.977
120 min post OGTT	42.23(25.79–71.12)	39.80(25.04–67.00)	43.15(28.58–65.87)	−0.01	0.013	0.701	0.473
**islet function**							
HOMA-β	107.23(80.42–148.39)	111.77(80.13–160.77)	104.97(80.83–154.93)	−0	0.011	0.958	0.953
IGI	13.86(9.10–22.42)	15.70(9.07–26.87)	14.54(7.69–23.60)	0.025	0.017	0.108	0.151
BIGTT-AIR	1864.9(1458.7–2619.9)	2067.4(1490.8–3041.1)	1903.1(1427.1–2782.6)	0.003	0.014	0.823	0.84
CIR	144.77(90.66–217.29)	161.68(95.22–256.91)	141.62(85.79–227.11)	0.015	0.015	0.287	0.33
**insulin resistance**							
HOMA-IR	2.18(1.70–3.22)	2.39(1.70–3.33)	2.31(1.72–3.27)	0.001	0.01	0.771	0.951
ISIc	0.072(0.038–0.134)	0.066(0.041–0.112)	0.068(0.042–0.109)	0.007	0.014	0.905	0.603
BIGTT-SI	1.7 ± 0.7	1.8 ± 0.7	1.8 ± 0.7	−0.01	0.03	0.819	0.831
**disposition indexes (DI)**							
DI 1	13719(9406–17982)	14463(10376–19329)	13312(9764–17872)	0	0.014	0.987	0.993
DI 2	62.00(40.69–92.88)	64.74(41.25–108.93)	59.79(34.63–102.58)	0.014	0.016	0.434	0.404

Note: IGI, the insulinogenic index; ISIc, Matsuda’s insulin sensitivity index; DI 1, BIGTT-AIR*BIGTT-SI; DI 2, CIR/HOMA-IR.

### CTLA-4 +49 A/G, but not CT60, has a significant association with IA-2A positive rate in T1D individuals

The distribution of the four antibody measurements is shown in Table S1. We also assessed the associations between these two SNPs and positivity of islet specific autoantibody in 1035 T1D patients, and time since diagnosis and disease duration were included as covariates in all statistical analysis. As shown in Table 3, a higher frequency of IA-2A positivity was observed among T1D patients with the susceptible GG genotype compared with AA (OR = 0.51, 95% CI: 0.30–0.84, p = 0.008). This effect was not found in relation to GADA, ZnT8A or IAA distributions (All P > 0.05). However, CTLA-4 CT60 had no association with any auto-antibody positive rate in T1D patients (All P > 0.05), as shown in Table S6.

**Table 3:** Antibody association for CTLA-4 +49 G/A in T1D patients

	Pos	Neg	OR (95% CI)	P value
**ZnT8A**				
A	225	298	0.84 [0.68, 1.03]	0.09
GG	249	259	1	—
GA	167	220	0.79 [0.61, 1.03]	0.08
AA	29	39	0.77 [0.46, 1.29]	0.32
**GADA**				
A	372	184	0.89 [0.72, 1.09]	0.26
GG	379	156	1	—
GA	268	138	0.80 [0.61, 1.05]	0.11
AA	52	23	0.93 [0.55, 1.57]	0.79
**IA-2A**				
A	235	319	0.78 [0.64, 0.95]	0.01**
GG	266	269	1	—
GA	185	219	0.85 [0.66, 1.11]	0.23
AA	25	50	0.51 [0.30, 0.84]	0.008**
**IAA**				
A	60	175	0.93 [0.66, 1.31]	0.69
GG	63	172	1	—
GA	48	129	1.02 [0.65, 1.58]	0.94
AA	6	23	0.71 [0.28, 1.83]	0.48

Note: For each autoantibody, the first row indicates the minor allele for which the odds ratio (OR) is estimated (95% confidence intervals (CI) is shown between brackets. For the genotypic association the most common homozygous group was taken as the reference. The association tests include age at T1D onset and duration as covariates. Numbers in parenthesis indicate the frequency of this genotype or allele. Pos, positive; Neg, negative; **P < 0.01.

### +49 A/G affects CTLA-4 expression in nTreg and aTreg subsets, but not sTreg or CD4 Teff

**Figure 2:**
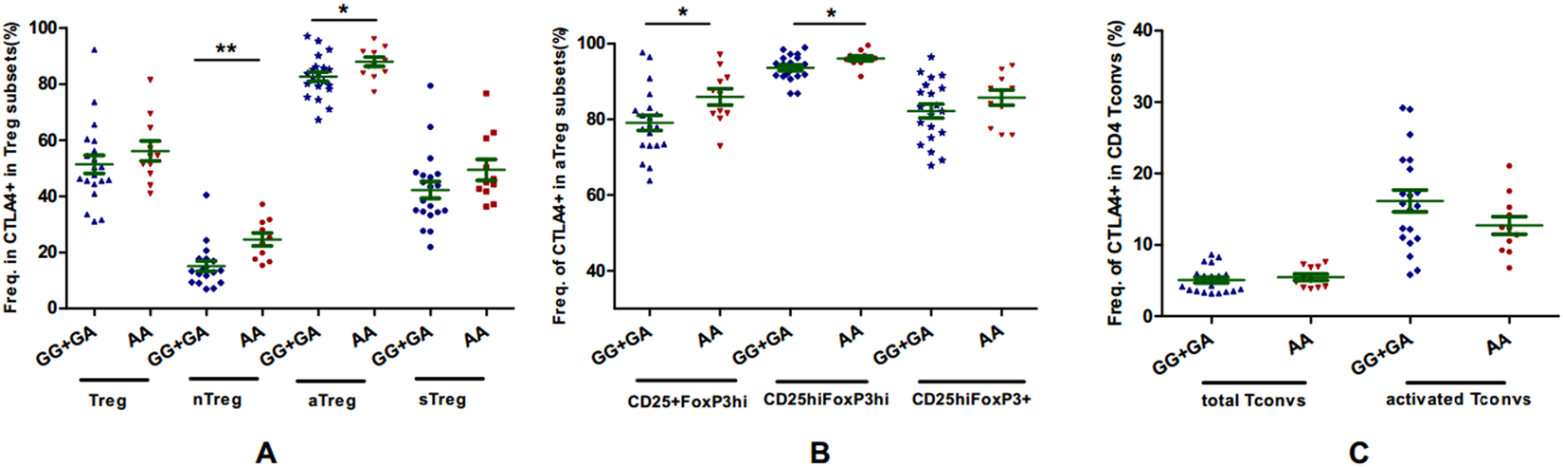
Effects of +49 G/A on CTLA-4 expression in CD4 Treg and Tconv cell subsets in healthy individuals. Note: (**A**–**C**) Effects of +49 G/A on intercellular CTLA-4 expression in CD4 Treg, activated Treg and CD4 Tconv cell subsets respectively. GG +GA = 20, AA = 11. *P < 0.05, **P < 0.01.

We further discerned the genetic contribution of +49 G/A in the absence of disease effects except for its association with humoral immunity in T1D patients. We assessed the effect of +49 G/A on CTLA-4 expression in a range of circulating T cell subtypes in an initial sample of 31 healthy individuals enrolled. As shown in Figure S4, by FACS analyses, we examined the intracellular CTLA-4 expression on three different T cell populations, including naïve/secreting/activated Tregs and naïve/memory CD4 Teff cells as determined by CD45RA, FOXP3 and CD25 expression, and naïve/CM/EM/TD CD8 T cells as determined by CD45RA and CCR7 expression. As shown in Fig. 2, samples from individuals with the CTLA-4 +49 GG/GA genotypes had significantly lower percentages of intracellular CTLA-4 positive naïve and activated Treg subsets (P = 0.0046 and 0.0317 respectively). Further analysis revealed that this SNP is significantly associated with CD25^+^FoxP3^hi^ and CD25^hi^FoxP3^hi^ activated Tregs (P = 0.0272 and 0.0174 respectively), but not CD25^hi^FoxP3^+^ activated Treg subsets. Interestingly, this SNP had no association with either secreting Tregs or memory CD4 Teff subsets (all P > 0.05). In addition, we did not detect an obvious CTLA-4 expression (frequency of most of the samples <1%) on naïve CD4 Teff cells or CD8 T cell subsets (naïve/CM/EM/TD), which were excluded from further analysis.

## Discussion

Studies from European descendents demonstrated that CT60 in the 3′-UTR of CTLA-4 gene was the most likely causal locus in susceptibility to autoimmune diseases, including type 1 diabetes^12,18,19^. CTLA-4 +49 G/A, a tagSNP for Asian population with moderate LD to CT60, is also a most studied SNP in genome studies^12,20–22^. Our results revealed that both +49 G/A and CT60 were associated with T1D risk in Chinese Han population, but +49 G/A displayed a more significant P value than CT60. The FPRP value of our case-control study remained below 0.2 even for a prior probability of 0.01. As previous studies on the association between +49 G/A and T1D risk are less powerful and still inclusive in Chinese Han population due to limited sample size, we further performed a meta-analysis combined our data with previous studies, the association between +49 G/A and T1D risk reached a significant GWAS P value. Taken together, these confirmed our results were quite robust.

In silico bioinformatics analysis indicated that both +49 G/A and CT60 may be functional with regulatory implications. Although they did not associate with eQTL in whole blood based on GTEx database, Westra HJ *et al*.^23^ reported that +49 G/A had significant cis-eQTL effects in whole blood (www.genenetwork.nl/bloodeqtlbrowser/), and Kasela S *et al*.^24^ reported both +49 G/A and CT60 had significant cis-eQTL effects in CD4+ and CD8+ T cells. Other studies further focused on the effects of +49 G/A on CTLA-4 expression in different T cell subsets, and found that the risk GG genotype of +49 G/A was associated with reduced cell surface CTLA-4 expression on activated T cells^25^, lower intracellular CTLA-4 protein level in CD4^+^ and CD4^+^ CD25^hi^ cells^26^ and decreased production of CTLA-4 protein^27^. In consequence, the G allele of +49 G/A confers a lower degree of inhibition of T-cell activity than the A allele, which may promote autoimmunity^28^. Here we found that lower intracellular CTLA-4 expression in naïve and activated CD4 Treg subsets from healthy individuals carrying GG or GA genotypes, which could be due to interference with the signal peptide of CTLA-4 leading to defects in the upregulation of CTLA-4 on the cell surface. Interestingly, our results further indicated that +49 G/A affected CTLA-4 expression in FoxP3^hi^, but not CD25^hi^Foxp3^+^ activated Treg subsets. Several studies also indicated that PHA or peptide of heat shock protein 60 induced a higher IFN-γ response, while GAD65 induced a lower IL-4 secretion in subjects with risk GG genotype of +49 G/A^29,30^, one of the interpretations might be individuals with G allele had lower CTLA-4 expression in Treg subsets and reduced suppressive function. It should be noted that CTLA-4 also expressed by diverse cell types, including activated CD4+ conventional T (Tconv) cells, activated B cells, and CD8 T cells^31–33^. But our results demonstrated that +49 G/A has no effect on CTLA-4 expression in activated CD4 Tconvs, and we did not find obvious CTLA-4 expression in either CD25^+^B cells or CD8 subsets from healthy individuals. Collectively, CTLA-4 expression in Treg subsets provide a potential mechanistic link between polymorphisms and the biological outcome of adaptive immune responses to self and to T1D pathogens.

Balic *et al*.^34^ found that the risk GG genotype or G allele of +49 G/A was associated with a younger age of onset and higher prevalence of positive anti-GADA. Another study also indicated that the G allele of this SNP had a significant association with risk of GADA and the GADA/IAA ratio^35^. As autoantibody positivity negatively correlated with time since diagnosis, levels of beta-cell antigens and immuno-inflammatory activity declined following diagnosis^36,37^. After adjustment for age at diagnosis and disease duration, we did not find any association between CT60 and positive rate of any islet-specific autoantibody, but we found that GG genotype or G allele of +49 G/A was significantly associated with IA-2A positivity in T1D patients. The possible explanation is that G allele of +49 G/A had lower CTLA-4 expression in Treg subsets, which might result in higher circulating follicular helper T cell frequencies and profoundly increased autoantibody production^38–40^. The discrepancy with previous studies might be due to diverse ethnicity, environmental, or other factors, population stratification, which needs confirmation by further studies.

Now accumulating evidence indicates that T1D genetic risk loci affected not only the immune system, but also β-cell function^41^. Moreover, GWAS central datasets^42^, generated by performing a meta-analysis of up to 21 GWAS informative for fasting glucose, fasting insulin and indices of β-cell function (HOMA-B) and insulin resistance (HOMA-IR) in up to 46,186 non-diabetic participants, demonstrated that for +49 G/A and CT60, only a significant association of +49 G/A was observed with fasting plasma glucose level (P = 0.01711). But in our study, we did not find any association between these two SNPs and insulin release or insulin sensitivity indexes in either healthy controls or newly diagnosed T1D patients, which suggested they might not affect β-cell function.

In conclusion, our results revealed that both +49 G/A and CT60 in CTLA-4 gene are associated with T1D risk in Chinese Han population. Although we do not find any significant association between these two SNPs and beta cell function in either healthy individuals or newly diagnosed T1D patients, using T1D as prototype autoimmune disease, we found that the risk G allele of +49 G/A, but not CT60, is significantly associated with a higher rate of IA-2A positivity. Furthermore, individuals carrying risk G allele of +49 G/A have reduced CTLA-4 expression in naïve and activated Treg subsets. It is important to note that multiple genetic variants may act in concert to modulate disease susceptibility and we cannot exclude that other genetic factors contribute to the genetic risk associated with CTLA-4, which needs further confirmation.

## Electronic supplementary material

Electronic Supplementary Information
