# Supplementary material for: CTLA-4 +49 G/A, a functional T1D risk SNP, affects CTLA-4 level in Treg subsets and IA-2A positivity, but not beta-cell function
Source: Sci Rep. 2018 Jul 4;8:10074. doi: 10.1038/s41598-018-28423-9 (PMC6031668; doi:10.1038/s41598-018-28423-9)
Supplement: Supplementary file 1 — Electronic Supplementary Information [file 41598_2018_28423_MOESM1_ESM.doc]

**CTLA-4 +49 G/A, a functional T1D risk SNP, affects CTLA-4 level in Treg subsets and IA-2A positivity, but not beta-cell function**

*Yang Chen^#^, Shu Chen^#^, Yong Gu^#^, Yingjie Feng, Yun Shi, Qi Fu, Zhixiao Wang, Yun Cai, Hao Dai, Shuai Zheng, Min Sun, Mei Zhang, Xinyu Xu, Heng Chen, Kuanfeng Xu*, Tao Yang**

*Department of Endocrinology, the First Affiliated Hospital of Nanjing Medical University, Nanjing China, 210029*

Table S1 Clinical characteristics of the study population

|  | **T1D** | **Control** |
| --- | --- | --- |
| **n** | 1,035 | 2,575 |
| **Age at draw** | 25.8±9.0 | 48.9±12.7 |
| **Gender (M/F)** | 510/525 | 1263/1312 |
| **Age at diagnosis** | 19.9±13.3 | - |
| **Duration** | 5.9±4.6 | - |
| **autoantibody positive** |  |  |
| **ZnT8A (%)** | 445 (46.2) | - |
| **GADA (%)** | 699 (68.8) | - |
| **IA-2A (%)** | 476 (46.9) | - |
| **IAA (%)** | 117 (26.5) | - |
| **C-Peptide level (pmol/L) ^※^** |  |  |
| **Fasting** | 212.2±182.4 | - |
| **30min** | 468.9±393.5 | - |
| **120min** | 540.5±472.7 | - |

Note: Age shows as mean±SD; M, male, F, female. **^※^**C-peptide level was from 283 newly diagnosed T1D patients in Chinese Han population.

**Table S2 Laboratory measurements and calculation of glycemic indexes**

| **Trait** | **Measurement or calculation** |
| --- | --- |
| HOMA-B | (20 × fasting serum insulin (pmol/l))/(fasting plasma glucose (mmol/l) - 3.5) |
| Insulinogenic index | (Serum insulin at 30min (pmol/l) - fasting serum insulin (pmol/l)) /(plasma glucose at 30min (mmol/l) - fasting plasma glucose (mmol/l)) |
| BIGTT-AIR* | exp[8.20+(0.00178 × fasting serum insulin (pmol/l))+ (0.00168 × serum insulin 30 min (pmol/l))- (0.000383 × serum insulin 120 min (pmol/l))-(0.314 × Fasting plasma glucose (mmol/l))-(0.109 × plasma glucose 30min (mmol/l))+(0.0781 × plasma glucose 120min (mmol/l))+(0.180 × sex)+(0.032 × BMI)] |
| CIR | ([serum insulin 30 min {pmol/l}/6.945] × 100)/(plasma glucose 30 min{mmol/l}×[plasma glucose 30 min{mmol/l}−3.89]) |
| HOMA-IR | (Fasting plasma glucose (mmol/l) × fasting serum insulin (pmol/l)) / 135 |
| ISI_Matsuda_ | (10,000/√(fasting plasma glucose × fasting serum insulin) ×(mean plasma glucose × mean serum insulin during OGTT)) |
| BIGTT-SI* | exp[4.90-(0.00402 × fasting serum insulin (pmol/l))-(0.000556 × serum insulin 30min (pmol/l))- (0.00127 × serum insulin 120min (pmol/l))-(0.152 ×Fasting plasma glucose (mmol/l))-(0.00871 × plasma glucose 30min (mmol/l))-(0.0373 ×plasma glucose 120min (mmol/l))-(0.145 ×sex)-(0.0376 ×BMI)] |
| DI 1 | BIGTT-AIR × BIGTT-SI |
| DI 2 | CIR /HOMA-IR |

* Sex (female=0, male=1); exp[ ] denotes the exponential function.

**Table S3 Studies included in the meta-analysis on the association between CTLA-4 +49 G/A and T1D risk**

| **Studies** | **Year** | **T1D** | | | | **Control** | | | | **P value (HWE)** | |
| --- | --- | --- | --- | --- | --- | --- | --- | --- | --- | --- | --- |
|  |  | **Total** | **GG** | **GA** | **AA** | **Total** | **GG** | **GA** | **AA** | **Cases** | **Controls** |
| **Gong CX et al** ^[S1]^ | 2007 | 39 | 22 | 14 | 3 | 52 | 32 | 17 | 3 | 0.714 | 0.713 |
| **Jin P et al** ^[S2]^ | 2015 | 402 | 182 | 194 | 26 | 482 | 169 | 241 | 72 | 0.006 | 0.354 |
| **Lee YJ et al** ^[S3]^ | 2000 | 253 | 150 | 85 | 18 | 91 | 37 | 45 | 9 | 0.222 | 0.377 |
| **Osei-Hyiaman D et al** ^[S4]^ | 2001 | 350 | 74 | 166 | 110 | 420 | 42 | 177 | 201 | 0.439 | 0.741 |
| **Song XQ et al** ^[S5]^ | 2012 | 108 | 73 | 25 | 10 | 100 | 45 | 39 | 16 | 0.002 | 0.138 |
| **This study** | 2017 | 1005 | 529 | 403 | 73 | 2532 | 1178 | 1086 | 268 | 0.753 | 0.996 |
| **Wang L et al** ^[S6]^ | 2001 | 90 | 23 | 54 | 13 | 84 | 10 | 42 | 32 | 0.041 | 0.499 |
| **Yang JJ et al** ^[S7]^ | 2006 | 34 | 23 | 8 | 3 | 71 | 32 | 28 | 11 | 0.102 | 0.253 |
| **Zhang SX et al** ^[S8]^ | 2005 | 82 | 36 | 42 | 4 | 86 | 20 | 48 | 18 | 0.059 | 0.278 |

**Supplementary References**

S1 Gong Chunxiu, Shen Kunling, Wu Di, Yan Chun. The Polymorphic of Cytotoxic T-Lymphocyte-Associated Antigen 4 of Patients with Autoimmune Poly-glandular Syndrome Ⅲ. Journal of Capital Medical University, 2007; 28(1): 788-789.

S2 Jin P, Xiang B, Huang G, Zhou Z. The association of cytotoxic T-lymphocyte antigen-4 + 49A/G and CT60 polymorphisms with type 1 diabetes and latent autoimmune diabetes in Chinese adults. J Endocrinol Invest, 2015; 38(2): 149-154.

S3 Lee YJ, Huang FY, Lo FS, Wang WC, Hsu CH, Kao HA, Yang TY, Chang JG. Association of CTLA4 gene A-G polymorphism with type 1 diabetes in Chinese children. Clin Endocrinol (Oxf), 2000; 52(2): 153-157.

S4 Osei-Hyiaman D, Hou L, Zhiyin R, Zhiming Z, Yu H, Amankwah AA, Harada S. Association of a novel point mutation (C159G) of the CTLA4 gene with type 1 diabetes in West Africans but not in Chinese. Diabetes, 2001; 50(9): 2169-2171.

S5 Song Xinqiang, Yi Caohui, Pan Li, Zhang Sihe. Association between CTLA-4 gene polymorphism and type 1 diabetes mellitus in Han population in the south of Henan province. Acta Uinversitis Medicals Anhui, 2012; 47(4): 431-434.

S6 Wang Luan, Yu Hongwei, Yan Shengli, Zhao Shihua, Wang Yangang, Wang Fei. The association of cytotoxic T lymphocyte-associated antigen 4 gene polymorphism with type 1 diabetes mellitus and autoimmune thyroid diseases in Chinese Han population. Chinese Journal of Endocrinology and Metabolism, 2001; 17(4): 228-231.

S7 Yang Jianjun, Zhang Yuhong, Huo Zhenghao, Zhu Xiaoquan, Song Liang, Yang Ze. An Analysis on Susceptible Genes Polymorphism of Insulin Dependent Diabetes Mellitus in Children in Ningxia. Journal of Ningxia Medical College, 2006; 28(5): 374-378.

S8 Zhang Shaowei, Ding Li, Li Pengfei, Qian Cong, Lan Jicheng, Zhao Wenzhou. Study on the association of CTLA-4 gene exon 1 49 G/A polymorphism with T1D patients and their parents. Natl Med J China, 2005; 85(11): 788.

**Table S4 Functional annotation of +49 G/A** and CT60 in CTLA-4 gene

| **SNP** | **Epigenome ID** | **Group** | **Description** | **H3K4me1** | **H3K4me3** | **H3K27ac** | **H3K9ac** |
| --- | --- | --- | --- | --- | --- | --- | --- |
| +49 G/A | E062 | Blood & T-cell | Primary mononuclear cells from peripheral blood | H3K4me1_Enh | H3K4me3_Pro | H3K27ac_Enh | H3K9ac_Pro |
| +49 G/A | E034 | Blood & T-cell | Primary T cells from peripheral blood | H3K4me1_Enh | H3K4me3_Pro | H3K27ac_Enh |  |
| +49 G/A | E045 | Blood & T-cell | Primary T cells effector/memory enriched from peripheral blood | H3K4me1_Enh | H3K4  me3_Pro | H3K27ac_Enh |  |
| +49 G/A | E033 | Blood & T-cell | Primary T cells from cord blood | H3K4me1_Enh | H3K4me3_Pro |  |  |
| +49 G/A | E044 | Blood & T-cell | Primary T regulatory cells from peripheral blood | H3K4me1_Enh | H3K4me3_Pro | H3K27ac_Enh |  |
| +49 G/A | E043 | Blood & T-cell | Primary T helper cells from peripheral blood | H3K4me1_Enh | H3K4me3_Pro | H3K27ac_Enh |  |
| +49 G/A | E039 | Blood & T-cell | Primary T helper naive cells from peripheral blood | H3K4me1_Enh | H3K4me3_Pro | H3K27ac_Enh |  |
| +49 G/A | E041 | Blood & T-cell | Primary T helper cells PMA-I stimulated | H3K4me1_Enh | H3K4me3_Pro | H3K27ac_Enh |  |
| +49 G/A | E042 | Blood & T-cell | Primary T helper 17 cells PMA-I stimulated | H3K4me1_Enh | H3K4me3_Pro | H3K27ac_Enh |  |
| +49 G/A | E040 | Blood & T-cell | Primary T helper memory cells from peripheral blood 1 | H3K4me1_Enh | H3K4me3_Pro | H3K27ac_Enh |  |
| +49 G/A | E037 | Blood & T-cell | Primary T helper memory cells from peripheral blood 2 | H3K4me1_Enh | H3K4me3_Pro | H3K27ac_Enh |  |
| +49 G/A | E048 | Blood & T-cell | Primary T CD8+ memory cells from peripheral blood | H3K4me1_Enh | H3K4me3_Pro | H3K27ac_Enh |  |
| +49 G/A | E038 | Blood & T-cell | Primary T helper naive cells from peripheral blood | H3K4me1_Enh | H3K4me3_Pro | H3K27ac_Enh | H3K9ac_Pro |
| +49 G/A | E047 | Blood & T-cell | Primary T CD8+ naive cells from peripheral blood | H3K4me1_Enh | H3K4me3_Pro | H3K27ac_Enh |  |
| +49 G/A | E046 | HSC & B-cell | Primary Natural Killer cells from peripheral blood | H3K4me1_Enh | H3K4me3_Pro |  |  |
| CT60 | E034 | Blood & T-cell | Primary T cells from peripheral blood | H3K4me1_Enh |  | H3K27ac_Enh |  |
| CT60 | E045 | Blood & T-cell | Primary T cells effector/memory enriched from peripheral blood | H3K4me1_Enh | H3K4me3_Pro | H3K27ac_Enh |  |
| CT60 | E033 | Blood & T-cell | Primary T cells from cord blood | H3K4me1_Enh |  |  |  |
| CT60 | E044 | Blood & T-cell | Primary T regulatory cells from peripheral blood | H3K4me1_Enh | H3K4me3_Pro | H3K27ac_Enh |  |
| CT60 | E043 | Blood & T-cell | Primary T helper cells from peripheral blood | H3K4me1_Enh |  | H3K27ac_Enh |  |
| CT60 | E039 | Blood & T-cell | Primary T helper naive cells from peripheral blood | H3K4me1_Enh | H3K4me3_Pro | H3K27ac_Enh |  |
| CT60 | E041 | Blood & T-cell | Primary T helper cells PMA-I stimulated | H3K4me1_Enh | H3K4me3_Pro | H3K27ac_Enh |  |
| CT60 | E042 | Blood & T-cell | Primary T helper 17 cells PMA-I stimulated | H3K4me1_Enh | H3K4me3_Pro | H3K27ac_Enh |  |
| CT60 | E040 | Blood & T-cell | Primary T helper memory cells from peripheral blood 1 | H3K4me1_Enh | H3K4me3_Pro |  |  |
| CT60 | E037 | Blood & T-cell | Primary T helper memory cells from peripheral blood 2 | H3K4me1_Enh | H3K4me3_Pro |  |  |

Note: black= missing data.

**Table S5 Associations of CTLA-4 CT60 With Quantitative Traits in 1280 Glucose-Tolerant East Chinese Individuals**

|  | **AA** | **GA** | **GG** | **β** | **SE** | **P** | **adjusted P** |
| --- | --- | --- | --- | --- | --- | --- | --- |
| n (men/women) | 840(274/566) | 337(93/244) | 89(29/60) |  |  |  |  |
| Age (years) | 55.46±8.95 | 56.86±9.78 | 54.55±9.51 |  |  |  |  |
| BMI (kg/m2) | 23.37±3.11 | 23.38±3.21 | 24.24±5.94 | 0.26 | 0.155 | 0.094 |  |
| **Plasma glucose (mmol/l)** |  |  |  |  |  |  |  |
| Fasting | 5.34±0.36 | 5.33±0.34 | 5.30±0.32 | -0.019 | 0.016 | 0.34 | 0.227 |
| 30 min post OGTT | 8.73±1.47 | 8.60±1.42 | 8.54±1.74 | -0.118 | 0.066 | 0.103 | 0.073 |
| 120 min post OGTT | 6.19±0.98 | 6.17±0.93 | 6.12±0.96 | -0.049 | 0.043 | 0.567 | 0.253 |
| **Serum insulin (mIU/l)** |  |  |  |  |  |  |  |
| Fasting | 9.74(7.28-13.48) | 10.26(7.33-14.51) | 9.01(6.73-13.16) | -0.043 | 0.31 | 0.893 | 0.889 |
| 30 min post OGTT | 56.40(37.72-88.76) | 62.50(41.00-98.65) | 50.34(37.92-72.67) | -0.425 | 2.47 | 0.831 | 0.863 |
| 120 min post OGTT | 42.71(27.44-64.37) | 40.76(25.32-71.52) | 36.03(23.20-69.72) | 1.35 | 2.09 | 0.309 | 0.518 |
| **islet function** |  |  |  |  |  |  |  |
| HOMA-β | 106.41(80.20-152.93) | 116.09(81.74-170.19) | 104.60(79.40-143.88) | 0.003 | 0.011 | 0.635 | 0.782 |
| IGI | 14.88(8.30-24.13) | 15.75(9.19-28.60) | 14.08(9.26-20.38) | 0.025 | 0.019 | 0.097 | 0.176 |
| BIGTT-AIR | 1941.9(1451.1-2799.9) | 2118.2(1498.6-3124.0) | 1896.1(1493.5 -2571.7) | -274.5 | 1115.8 | 0.801 | 0.806 |
| CIR | 143.5(86.6-227.9) | 162.41(96.89-269.10) | 145.53(93.86-210.12) | 0.014 | 0.017 | 0.287 | 0.408 |
| **insulin resistance** |  |  |  |  |  |  |  |
| HOMA-IR | 2.31(1.70-3.24) | 2.44(1.74-3.46) | 2.12(1.56-3.13) | -0.004 | 0.011 | 0.941 | 0.696 |
| ISIc | 0.068(0.042-0.108) | 0.062(0.038-0.113) | 0.081(0.047-0.153) | 0.011 | 0.015 | 0.878 | 0.456 |
| BIGTT-SI | 7.02±3.45 | 6.84±3.78 | 7.51±3.85 | 0.073 | 0.162 | 0.67 | 0.653 |
| **disposition indexes (DI)** |  |  |  |  |  |  |  |
| DI 1 | 13475(9966-181557) | 14336(9611-19264) | 14869 (9898-19575) | -0.001 | 0.015 | 0.999 | 0.935 |
| DI 2 | 60.36(36.20-102.65) | 64.72(40.92-109.11) | 65.22(41.37-102.27) | 0.021 | 0.018 | 0.219 | 0.235 |

**Table S6 Antibody association for CTLA-4 CT60 in T1D patients**

|  | **Pos** | **Neg** | **OR (95% CI)** | **P value** |
| --- | --- | --- | --- | --- |
| **ZnT8A** |  |  |  |  |
| A | 133 | 165 | 0.91 [0.71, 1.16] | 0.44 |
| GG | 326 | 361 | 1 | - |
| GA | 111 | 137 | 0.90 [0.67, 1.20] | 0.47 |
| AA | 11 | 14 | 0.87 [0.39, 1.94] | 0.73 |
| **GADA** |  |  |  |  |
| A | 206 | 109 | 0.84 [0.65, 1.08] | 0.18 |
| GG | 511 | 219 | 1 | - |
| GA | 168 | 91 | 0.79 [0.59, 1.07] | 0.13 |
| AA | 19 | 9 | 0.90 [0.40, 2.03] | 0.81 |
| **IA-2A** |  |  |  |  |
| A | 135 | 179 | 0.83 [0.65, 1.06] | 0.13 |
| GG | 350 | 381 | 1 | - |
| GA | 119 | 139 | 0.93 [0.70, 1.24] | 0.63 |
| AA | 8 | 20 | 0.44 [0.19, 1.00] | 0.051 |
| **IAA** |  |  |  |  |
| A | 34 | 93 | 1.02 [0.67, 1.56] | 0.92 |
| GG | 86 | 240 | 1 | - |
| GA | 28 | 79 | 0.99 [0.60, 1.63] | 0.97 |
| AA | 3 | 7 | 1.20 [0.30, 4.73] | 0.8 |

Note: For each autoantibody, the first row indicates the minor allele for which the odds ratio (OR) is estimated (95% confidence intervals (CI) is shown between brackets. For the genotypic association the most common homozygous group was taken as the reference. The association tests include age at T1D onset and duration as covariates. Numbers in parenthesis indicate the frequency of this genotype or allele. Pos, positive; Neg, negative; **, P<0.01.

Potentially relevant articles identified and screened for retrieval (**n=256**)

Studies excluded following review of abstracts (**n=245**)

Studies retrieved for more detailed evaluation (**n=11**)

three studies were ineligible for the following reasons:

two studies were duplicated data,

one studies were deviated from HWE (*P* <0.05).

Data from **8** published articles and this study consider for inclusion in meta-analysis

**Figure S1 Systematic review flow diagram.** We performed an exhaustive search on studies that examined the association of CTLA-4 +49 G/A polymorphism with T1D risk. Data were collected from the *PubMed, Embase, Web of Science, CBMdisc* and *CNKI* databases and completed on Jan, 2018. n, number of studies.


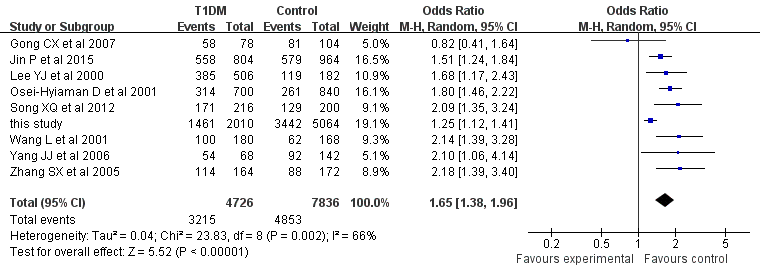


**Figure S2 Stratified analysis pooled ORs and 95% CI for the association between CTLA-4 +49 G/A and T1D risk.** The area of the squares reflects the study-specific weight..

**A B C**

**Figure S3 Associations of CTLA-4 CT60 with fasting C-peptide, C-peptide AUC and residual C-peptide in newly diagnosed T1D patients.** Note: **A-C,** Association between CTLA-4 CT60 and fasting C-peptide, C-peptide AUC, and residual C-peptide respectively. GG=207, GA=71, AA=5; fasting C-peptide and AUC were adjusted by age, gender, age at T1D onset and duration; residual C-peptide was defined as Fasting C-peptide≥ 0.2nmol/L.

**A**

**B**

**C**

**Figure S4 Representative flow cytometry graphs show frequency and CTLA-4 expression of CD4 Treg and Tconv cell subsets. A**, Representative flow cytometry graphs gated for CD4 Treg subsets. Treg, FoxP3^+^CD25^+^; nTreg, CD45RA^+^ Treg; aTreg, CD45RA^-^FoxP3^hi^ or CD45RA^-^CD25^hi^; sTreg, CD45RA^-^FoxP3^+^CD25^i^; **B**, Representative flow cytometry graphs gated for CTLA-4 expression in CD4 Treg subsets. From left to right: Expression of CTLA4 in total Treg, nTreg, aTreg and sTreg respectively (%); **C**, Representative flow cytometry graphs gated for CTLA-4 expression in CD4 Tconv cells.
